# Estimation of skin surface roughness *in vivo* based on optical coherence tomography combined with convolutional neural network

**DOI:** 10.3389/fmed.2024.1453405

**Published:** 2024-10-11

**Authors:** Zhiqun Zhang, Zhida Chen, Zhenqian Li, Jian Zou, Jian Guo, Kaihong Chen, Yong Guo, Zhifang Li

**Affiliations:** ^1^The Internet of Things and Artificial Intelligence College, Fujian Polytechnic of Information Technology, Fuzhou, Fujian, China; ^2^Key Laboratory of Optoelectronic Science and Technology for Medicine, Ministry of Education, Fujian Provincial Key Laboratory of Photonics Technology, Fujian Provincial Engineering Technology Research Center of Photoelectric Sensing Application, College of Photonic and Electronic Engineering, Fujian Normal University, Fuzhou, Fujian, China

**Keywords:** skin roughness, optical coherence tomography, convolutional neural network, epidermal thickness, attenuation coefficient

## Abstract

The texture of human skin is influenced by both external and internal factors, and changes in wrinkles can most directly reflect the state of the skin. Skin roughness is primarily used to quantify the wrinkle features of the skin. Therefore, effective and accurate quantification of skin roughness is essential in skincare, medical treatment, and product development. This study proposes a method for estimating the skin surface roughness using optical coherence tomography (OCT) combined with a convolutional neural network (CNN). The proposed algorithm is validated through a roughness standard plate. Then, the experimental results revealed that skin surface roughness including arithmetic mean roughness and depth of roughness depends on age and gender. The advantage of the proposed method based on OCT is that it can reduce the effect of the skin surface’s natural curvature on roughness. In addition, the method is combined with the epidermal thickness and dermal attenuation coefficient for multi-parameter characterization of skin features. It could be seen as a potential tool for understanding the aging process and developing strategies to maintain and enhance skin health and appearance.

## 1 Introduction

With the global increase in the aging population, research on age-related alterations of skin is receiving growing interest (1). The passage of time and repeated exposure to UV radiation are the two main factors for aged skin. As age advances, there is a gradual loss of collagen in the skin, resulting in the development of wrinkles (2). Simultaneously, exposure to UV radiation can cause skin dryness, abnormal pigmentation, and other issues, ultimately leading to the formation of wrinkles on the skin (3). Quantifying skin wrinkles is of significant importance in the fields of skincare, medical treatment, and product development (4, 5).

The quantification of skin wrinkles allows for objective assessment of wrinkle severity, enabling accurate evaluation of treatment efficacy and product performance. Various methods are used to quantify wrinkles, including both subjective and objective approaches. Subjective methods involve visual assessments by trained professionals or self-assessments by individuals themselves. These methods rely on scoring systems (five grades and nine grades) to evaluate the depth, length, and overall appearance of wrinkles (6, 7). However, subjective scoring relies more on individuals’ subjective judgments and perceptions and often fails to capture minor changes.

In addition, objective methods utilize advanced imaging technologies and computer analysis to provide precise and quantitative measurements of wrinkle parameters. These methods can be divided into two-dimensional (2D) camera approaches and three-dimensional (3D) scanning techniques. Two-dimensional approaches for assessing skin include the use of mobile phone cameras with natural light sources (8), charge-coupled device (CCD) cameras utilizing UVA light sources (9), and speckles with laser light sources (10). However, two-dimensional photograph-based analyses by observers are vulnerable to noise, variable magnifications, and surrounding illumination. Furthermore, speckle contrast does not directly measure the height fluctuation of the skin surface. Three-dimensional scanning techniques contain 3D stereophotogrammetry (5) and phaseshift rapid *in vivo* measurement of the skin (PRIMOS) (11–13). However, motion artifacts during the image capture process in 3D stereophotogrammetry and PRIMOS can introduce errors, making it difficult to provide accurate and reliable measurements of skin roughness (14).

Optical coherence tomography (OCT) can overcome the above problems by providing non-invasive, real-time, and high-resolution imaging of the skin (15, 16). Surface roughness measurement based on OCT was proposed to assess the arithmetic mean roughness and average depth of roughness (17, 18). The roughness estimation was calculated based on the height relative to the central line of best fit through the dermal–epidermal junction (DEJ) (17). However, the central line of the skin surface differs from that defined by the International Organization for Standardization (ISO), which is based on the mean of height fluctuations (19). Additionally, image processing techniques such as the Gaussian filter, median filter, and differential filter were used to extract the ideal skin surface boundary (18). However, it is difficult for all skin since some empirical parameters in these image processing algorithms.

In this study, the method of OCT combined with the U-Net architecture of a convolutional neural network (CNN) is proposed for the evaluation of skin surface roughness using the advantages of 3D imaging and accurate boundary location. This choice is driven by the advantages of U-Net, namely, its ability to provide effective segmentation results and its limited requirement for training data. In this study, Section 2 introduces the OCT system, the accurate location of skin surface based on CNN, and the definition of arithmetic mean roughness and the depth of roughness. Section 3 first validates the algorithm using a roughness standard plate and explores the function of skin surface roughness in terms of age and gender. Section 4 offers a discussion of the findings and analyzes the strengths of the proposed methodology.

## 2 Materials and methods

### 2.1 Optical coherence tomography (OCT)

**Figure 1:**
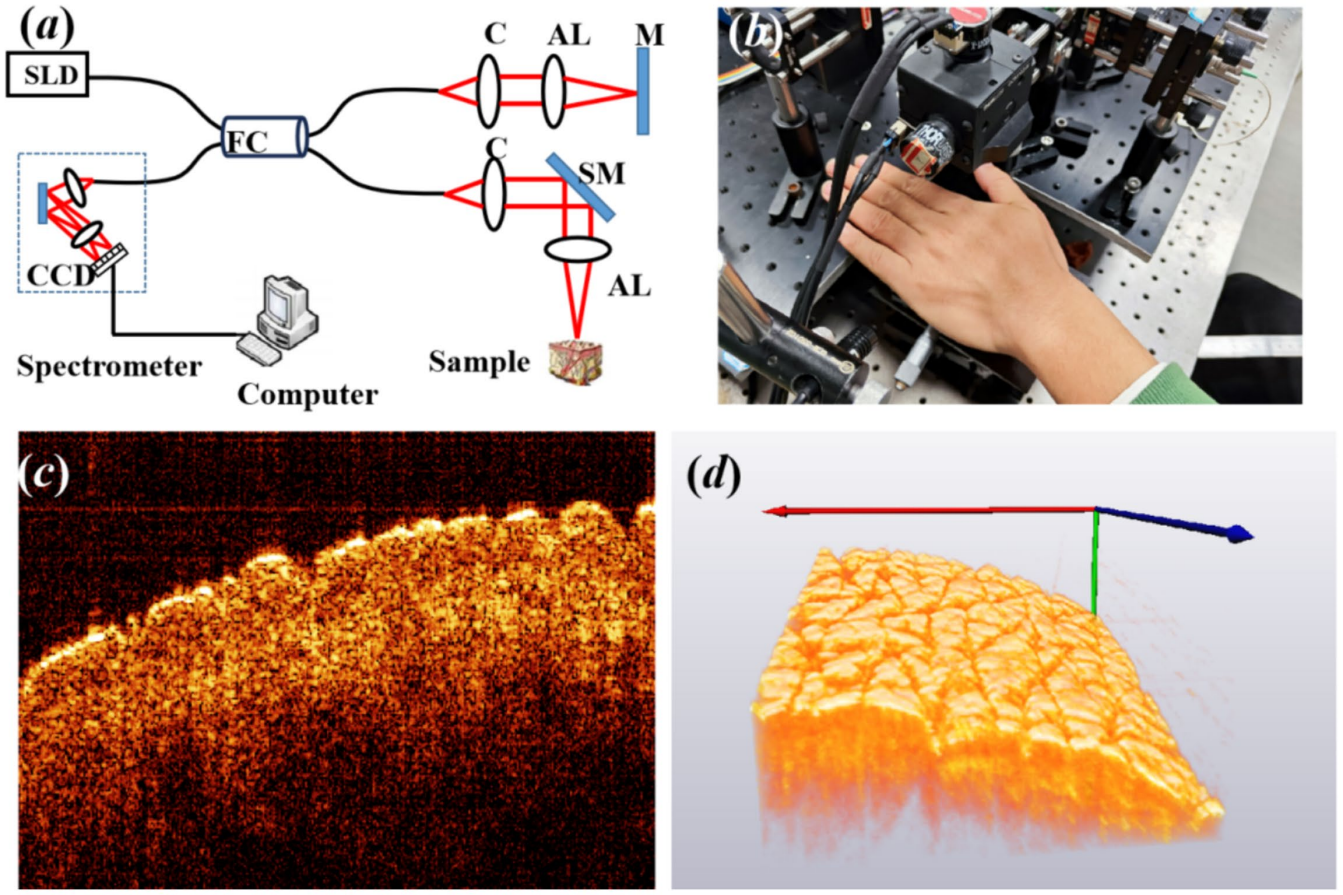
**(A)** Experimental setup of OCT, where SLD is the light source of the superluminescent diode, FC is fiber coupler, C is collimator, AL is achromatic lens, M is mirror, and SM is scanning mirror. **(B)** The back of the left hand for imaging, **(C)** typical cross-sectional OCT image, and **(D)** three-dimensional (3D) OCT image of the back of the left hand.

A schematic of our spectral domain optical coherence tomography (SD-OCT) system is illustrated in Figure 1A. The light source is a 12-mW superluminescent diode (SLD) with an FWHM bandwidth of 85 nm centered at 1310 nm (S5FC1021P, Thorlabs, Newton, NC, United States). Light is transmitted into a fiber coupler (FC) and then split into reference (50%) and sample (50%) arms, where collimators are used to obtain collimated light. A galvo scanning mirror (SM) and an achromatic lens (AL) with a focal length of 50 mm make up the scanning structure. The axial and lateral resolutions of the system in air are approximately 8.9 μm and 18.2 um, respectively. The detection arm consists of a spectrometer with a single line-scan camera (C-1235-1385, Wasatch Photonics, Logan, UT, United States) to construct a 3D image, resulting in the acquisition of 400 cross-sectional OCT images with a beam position increment of 25 μm.

A total of 16 volunteers were recruited for the experiment, including nine male individuals and seven female individuals. At the time of enrollment, subjects’ ages ranged between 15 and 45 years, and all volunteers had no smoking history. Prior to the experiment, all volunteers signed an informed consent form, indicating their understanding and agreement to participate in the study. Before the imaging procedure, the region of interest of the skin was marked, washed using a cleansing cream, and exposed to a constant temperature and humidity in order to stabilize the experimental conditions. Subsequently, the volunteer was asked to place the back of the left hand on the designated area of the collection platform, as shown in Figure 1B, maintaining a fixed and comfortable posture. The collection platform was designed to support the hand and minimize any possible movement or vibration, ensuring the accuracy of data collection. Figures 1C,D show the typical cross-sectional and 3D OCT image of the back of the left hand. The texture of skin wrinkles is shown in Figure 1D. All the research procedures using human participants were carried out at Fujian Normal University with approval from the Institutional Review Board for the Protection of Human Subjects in Research (IRB).

### 2.2 Detecting boundary of skin surface using CNN

**Figure 2:**
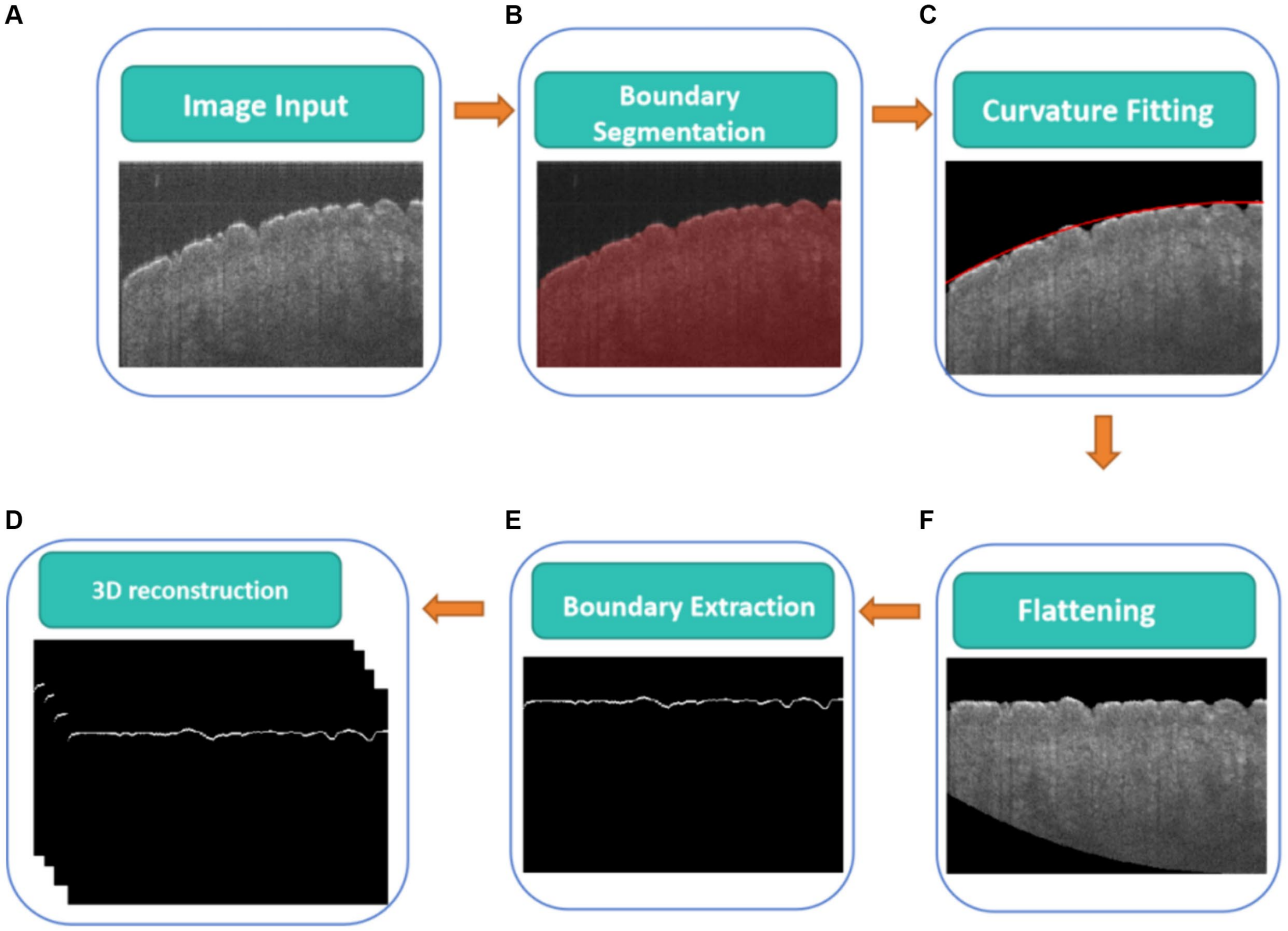
CNN-based algorithm for detecting boundary of skin surface, **(A)** original cross-sectional OCT images, **(B)** real boundary segmentation based on U-Net, **(C)** curvature fitting of real boundary height, **(D)** the flattening fitting boundary, **(E)** real boundary extraction on the flattening fitting boundary correction, **(F)** 3D real boundary.

Figure 2 illustrates a flowchart of a CNN-based algorithm for detecting the boundary of the skin surface including boundary segmentation, curvature fitting, flattening, and boundary extraction, which will be described in detail in the following paragraph.

Before measuring skin roughness, it is necessary to segment the boundaries of the skin surface and flatten the skin surface. Figure 2 shows a CNN-based algorithm for detecting the real boundary of the skin surface. The skin surface was segmented and detected using a CNN (Figure 2B), specifically employing the U-Net architecture proposed by Ronneberger et al. (20), which has been widely used for biological image segmentation (21, 22). Meanwhile, ResNet50 was used as the backbone feature extraction network (23). The Adam optimizer was used to update the model, allowing the network to automatically adjust the learning rate for each parameter based on its update history (24). The learning rate (LR) for this experiment was set at 0.0001, which directly affected the speed and performance of the training process (25). A loss function of 0.01 quantified the error between actual values and predicted values (26). Mean Intersection over Union (MIoU) was used to evaluate the accuracy of the image segmentation model (27).

**Figure 3:**
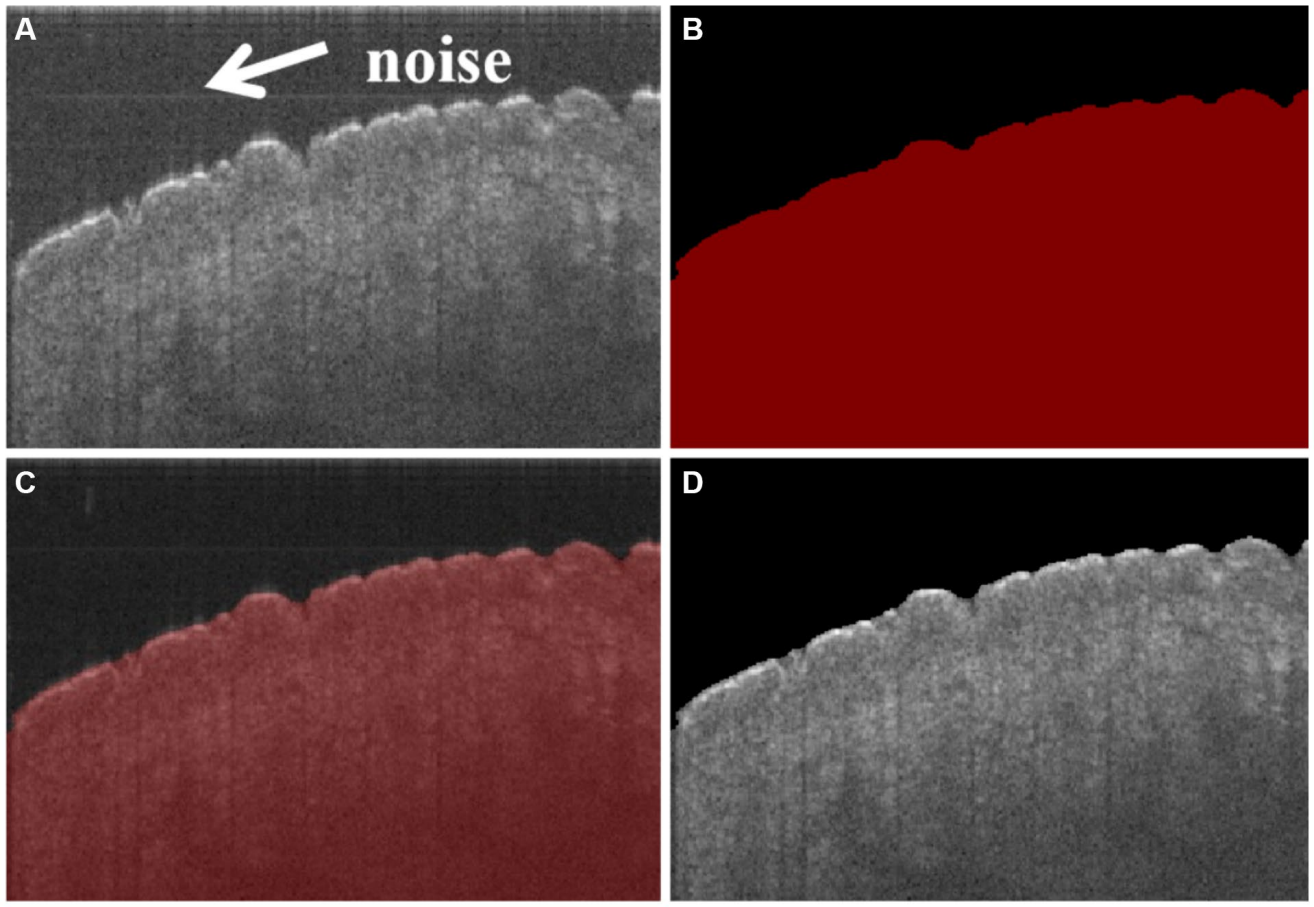
**(A)** Original cross-sectional OCT image of the skin, in which there is noise in the position of arrows, **(B)** masked image of skin segmentation based on CNN, **(C)** masked image superimposed with the original image, and **(D)** segmented image of the skin, in which the noise has been reduced comparing with **(A)**.

In the experiment, a total of 16 sets of data were collected, amounting to 6,400 samples. Among these samples, 1,600 were annotated using Labelme for the boundaries of the skin. Afterward, the annotated dataset was typically divided into a training set and a test set in a 9:1 ratio. The training batch size was set at 8, and the number of iterations was set at 100. An MIoU score of 98.36 indicated a high degree of similarity between the model’s predictions and the manual annotations, indicating a strong segmentation performance. In addition, Figure 3D shows that the noise in Figure 3A can be effectively reduced. It suggests that the model has successfully learned to extract the boundaries of the skin accurately, as shown in Figure 3, which lays a solid foundation for subsequent operations or tasks.

**Figure 4:**
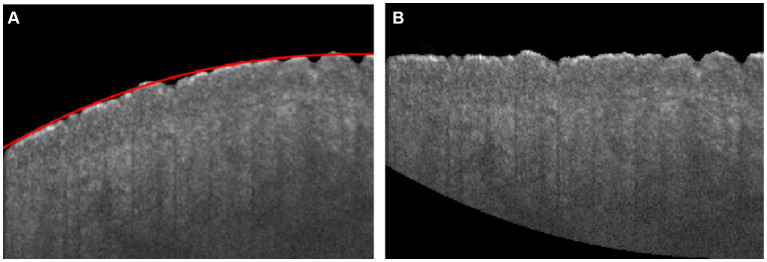
**(A)** Skin boundary curvature fitting, in which the red curve is the fitting boundary of skin; **(B)** Flattening boundary according to the fitting curve.

The boundary of the skin surface can be recorded based on the segmented image. However, the skin surface exhibits natural curvature, which can affect the assessment of roughness. Therefore, when calculating roughness, it is necessary to eliminate the influence of natural curvature. In this algorithm, the influence of natural curvature can be addressed by using the method of second-order polynomial fitting based on the least square method to find the curvature of the natural curvature in that region, as shown in Figure 2C. The flattening fitting boundary is shown in Figure 2D. Figure 4A demonstrates the fitting result of the skin. Subsequently, the curvature of the skin was flattened, as shown in Figure 4B, in which the fitting height of the boundary was set to the same height.

**Figure 5:**
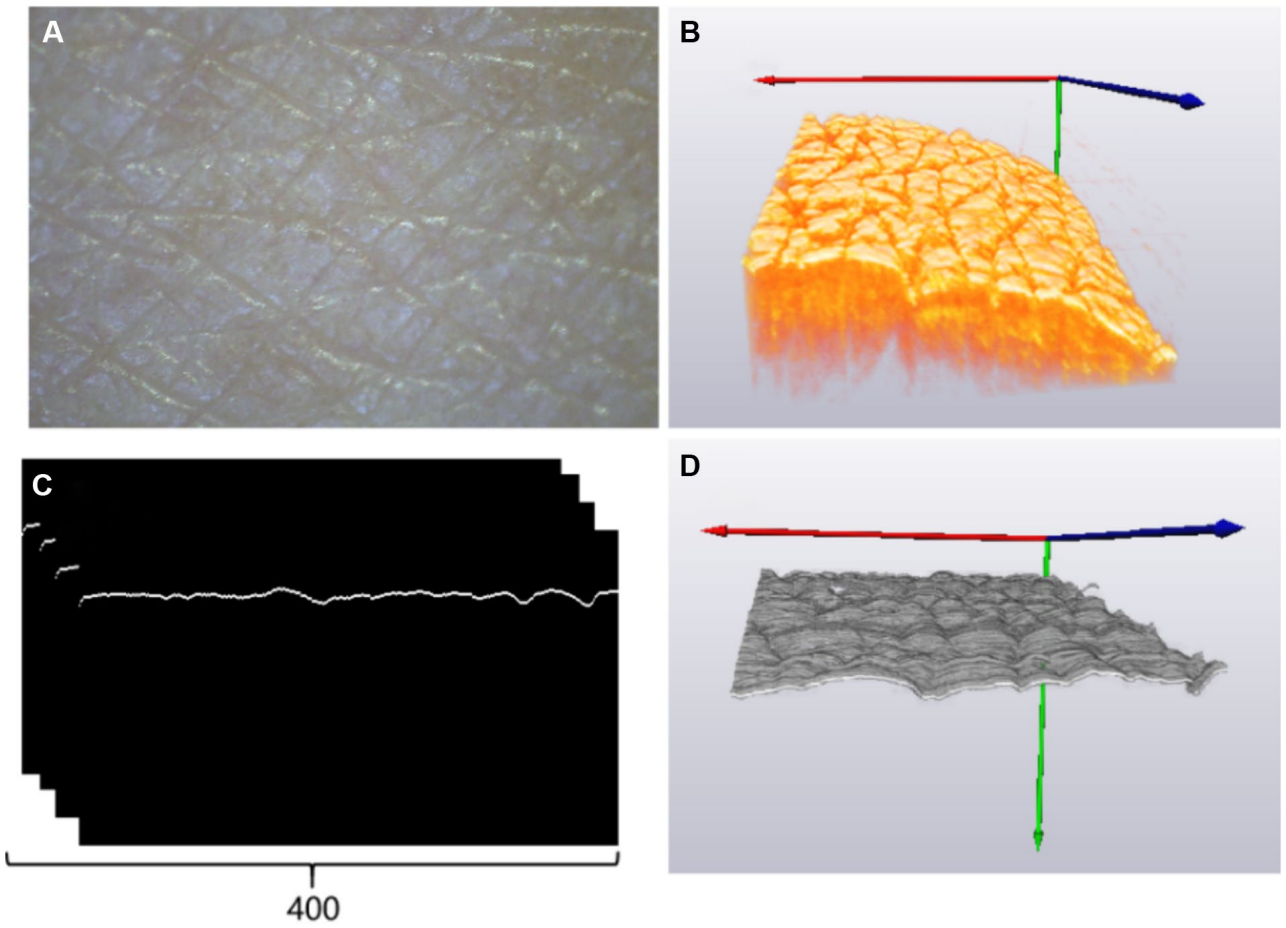
**(A)** Skin image of the back of the left hand under a stereomicroscope; **(B)** three-dimensional OCT image reconstruction results; **(C)** rear skin boundary; **(D)** three-dimensional skin boundary.

Once the acquisition of a cross-sectional skin boundary image was complete, the algorithm for 3D images of the skin surface was repeated to establish a three-dimensional (3D) topographic map of the skin, as shown in Figure 2F, and calculate 3D roughness data. Observations of the human skin surface under a stereomicroscope and OCT are shown in Figures 5A,B, respectively. Figure 5C shows a set of 400 B-scan images after segmenting the boundaries of the skin surface and flattening the skin surface. Figure 5D reveals the 3D reconstruction of Figure 5C, and the parameters of roughness were calculated based on Figure 5D. Figure 5A shows the skin roughness based on image texture, and Figure 5D shows the skin roughness according to the height, which is clearer than Figure 5A.

### 2.3 Quantification of surface roughness

According to the ISO 25178 standard established by the International Organization for Standardization (ISO), which is used for surface texture measurement, a series of surface texture parameters were defined to describe the morphology characteristics of a surface. Based on roughness standards and specific requirements, the arithmetic mean roughness ( $R_{a}$ ) and the depth of roughness ( $R_{z}$ ) were used for skin roughness. Their definitions are the arithmetic average of the absolute values of the surface height ( z ) and the maximum height between the highest peak and the lowest valley from the mean line within the measured region, respectively. The specific expressions of $R_{a}$ and $R_{z}$ are given as follows (19):


(1) $$R_{a}=\frac{1}{n_{x}\times n_{y}}\overset{n_{x}}{\underset{i=1}{\sum }}\overset{n_{y}}{\underset{j=1}{\sum }}|z\left(x_{i},y_{i}\right)|\text{,}$$

(2) $$R_{z}=\max\left(z\right)-\min\left(z\right)\text{,}$$

where $x_{i}$ and $y_{i}$ are two-dimensional spatial coordinates, respectively. Base on Equation 1, the arithmetic mean roughness ( $R_{a}$ ) provides an overall measure of the surface roughness. Moreover, using Equation 2, the depth of roughness ( $R_{z}$ ) indicates the maximum height variation on the surface. Both parameters including arithmetic mean roughness ( $R_{a}$ ) and the depth of roughness ( $R_{z}$ ) are related to the height fluctuation of the skin surface; thus, they depend on the axial resolution of OCT.

### 2.4 Statistical analysis

Correlation analysis was performed using Pearson’s correlation coefficients. To test validity, the roughness parameters of $R_{a}$ and $R_{z}$ were compared to the age (Pearson’s correlation). A Pearson correlation coefficient greater than 0.6 was considered a strong positive correlation.

## 3 Results

### 3.1 Validating the algorithm using a roughness standard plate

**Figure 6:**
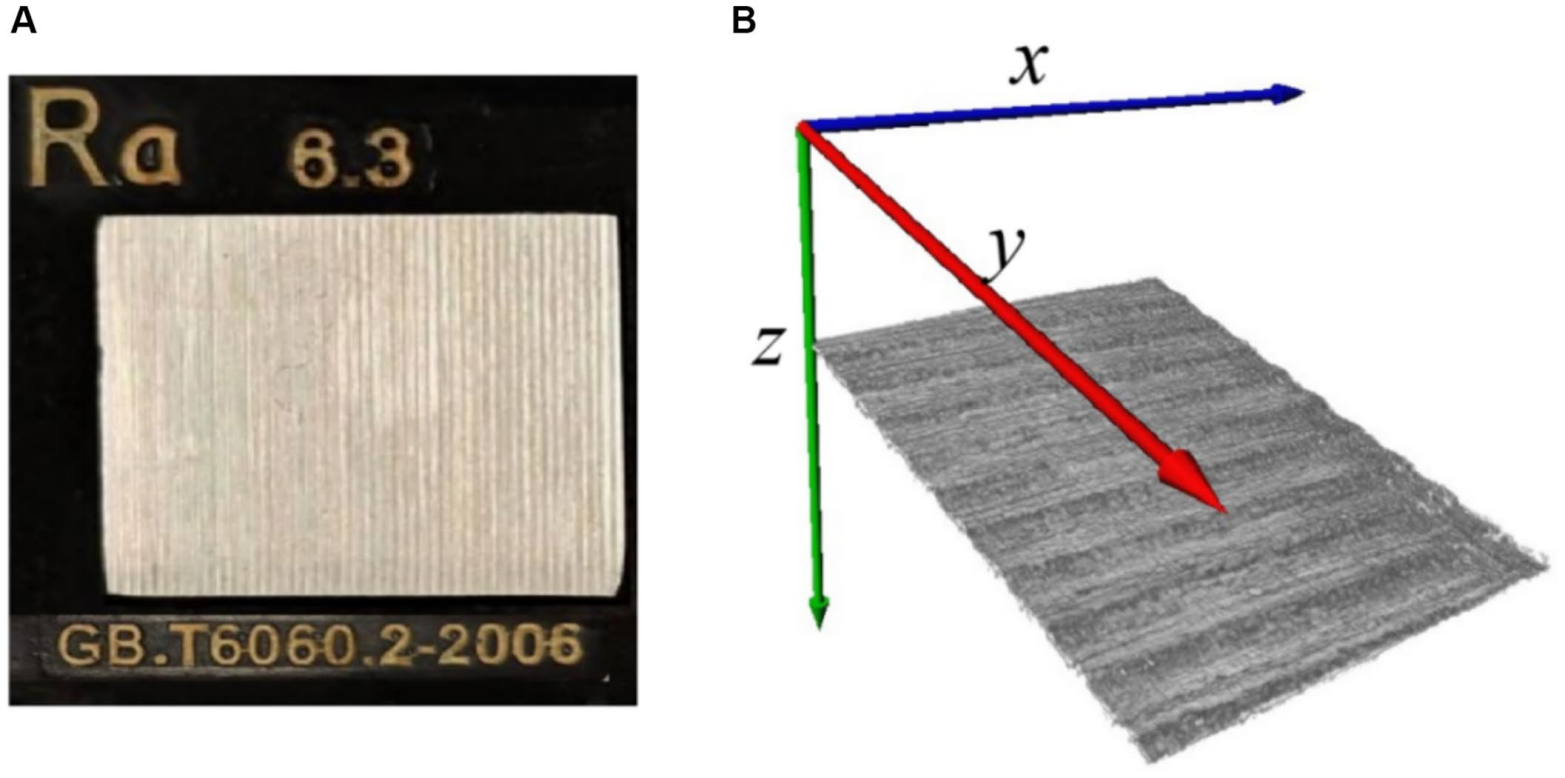
**(A)** Roughness standard plate; **(B)** 3D OCT image reconstruction of roughness standard plate.

First, the proposed algorithm for skin roughness was validated using a roughness standard plate, which was purchased from Dongguan Tangxia Aiceyi Electronic Instrument Trading Company, as shown in Figure 6A. Figure 6A shows the roughness standard plate with an arithmetic mean roughness *R_a_* of 6.3 μm, which complies with the GB.T6060.2–2006 standard. Figure 6B indicates 3D OCT images of the corresponding roughness standard plate, as shown in Figure 6A. Table 1 shows the arithmetic mean roughness *R_a_* on three positions of the roughness standard plate and demonstrates that the calculated value based on OCT is consistent with the standard defined in GB.T6060.2–2006. Thus, the proposed methods for roughness based on 3D OCT images provided an accurate and reliable measurement of roughness.

**Table 1:** Calculated arithmetic mean roughness of the three positions based on the proposed method is consistent with the standard value in GB.T6060.2–2006.

No.	Proposed method (μm)	Standard value (μm)	Error
1	6.47		2.7%
2	6.17	6.3	−2.1%
3	6.39		1.4%

### 3.2 Skin surface roughness dependent on age

**Figure 7:**
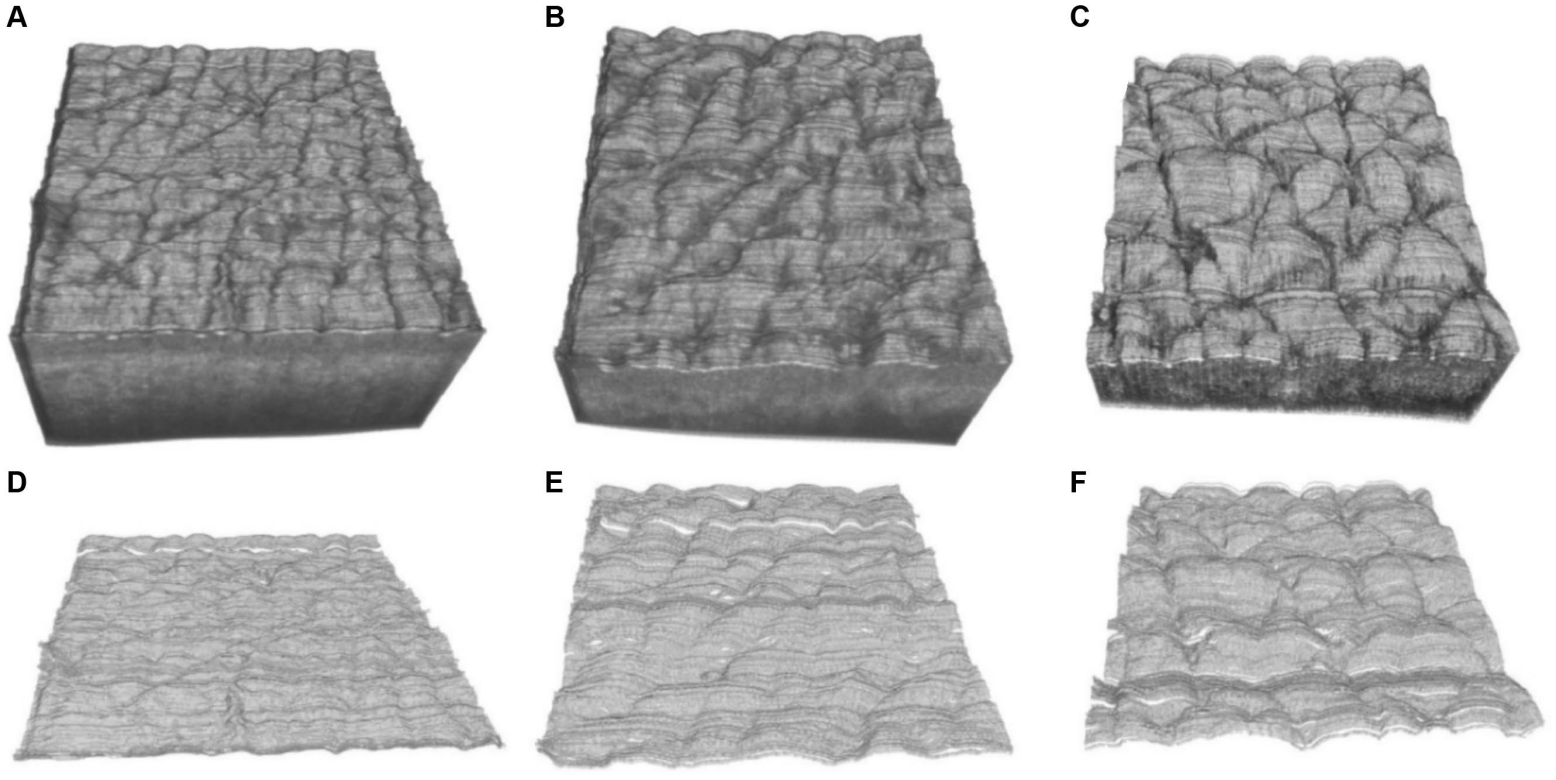
Three-dimensional OCT images at the ages of **(A)** 17, **(B)** 29, **(C)** 42 years, and **(D–F)** are the corresponding three-dimensional boundary images of **(A–C)**.

**Figure 8:**
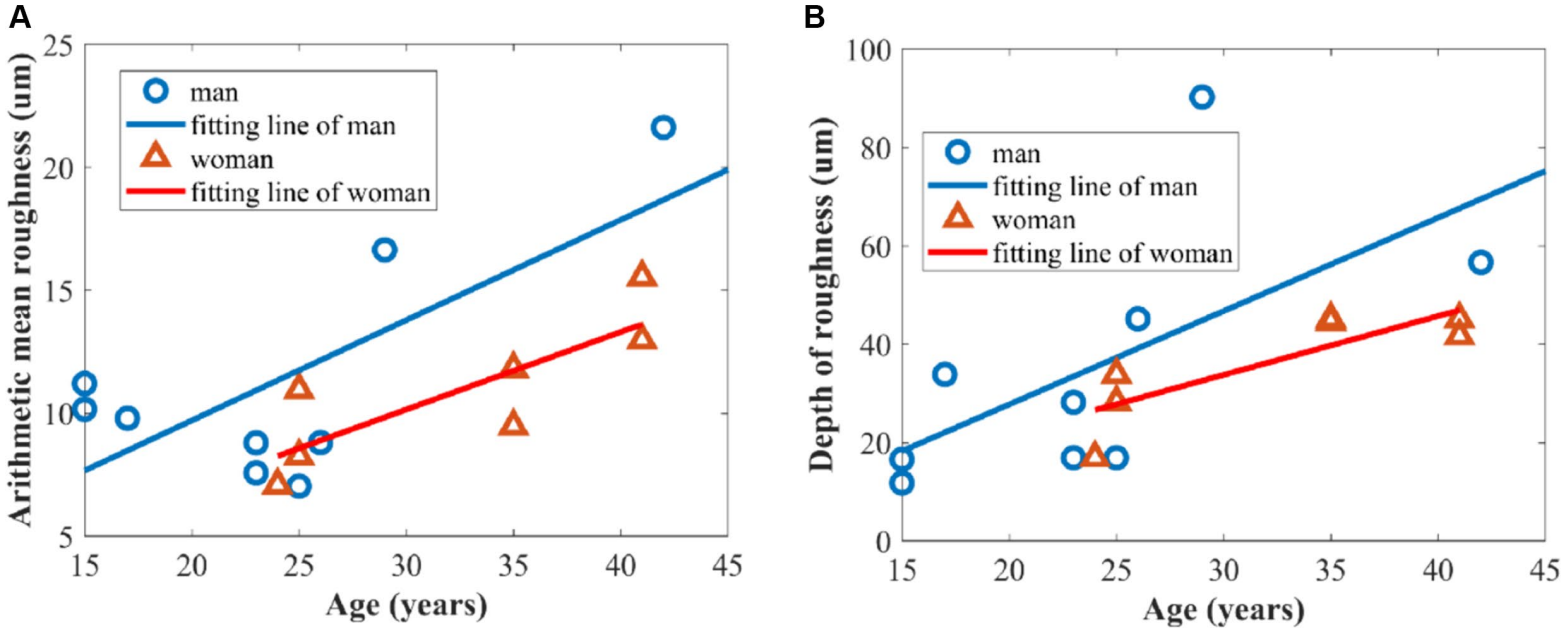
Relationship between skin roughness parameters and age of volunteers: **(A)** arithmetic mean roughness *R*_a_; **(B)** depth of roughness *R*_z_.

Figures 7A–C show the three-dimensional OCT images of the back of the hand’s skin, illustrating how the skin surface flattens with age. The texture of the skin surface, as observed in these OCT images, depends on age. To quantify the texture, we utilized *R*_a_ and *R*_z_ to explore the function of the age based on the three-dimensional skin boundary images, as shown in Figures 7D–F. Higher *R*_a_ values, shown in Figure 8A, indicate increased roughness, while higher values of *R*_z_ in Figure 8B indicate deeper depths of roughness.

Figure 8A shows a significant positive correlation between age and the arithmetic mean roughness in which Pearson’s correlation coefficients of men and women are 0.717 and 0.821, respectively. Meanwhile, there is a positive correlation between depth of roughness and age in Figure 8B, with Pearson’s correlation coefficients of 0.626 and 0.833, respectively, for men and women. This can be attributed to the gradual loss of collagen, which leads to a decrease in elasticity and firmness in the skin. In addition, the slowing down of epidermal cell turnover is also a significant contributing factor to increased skin roughness (28, 29).

Figure 8 also demonstrates that the overall roughness levels, as indicated by the two parameters of arithmetic mean roughness *R*_a_ and depth of roughness *R*_z_, were higher in men than in women over the age of 25 years old because women generally place more emphasis on skincare compared to men (30, 31).

## 4 Discussion

**Figure 9:**
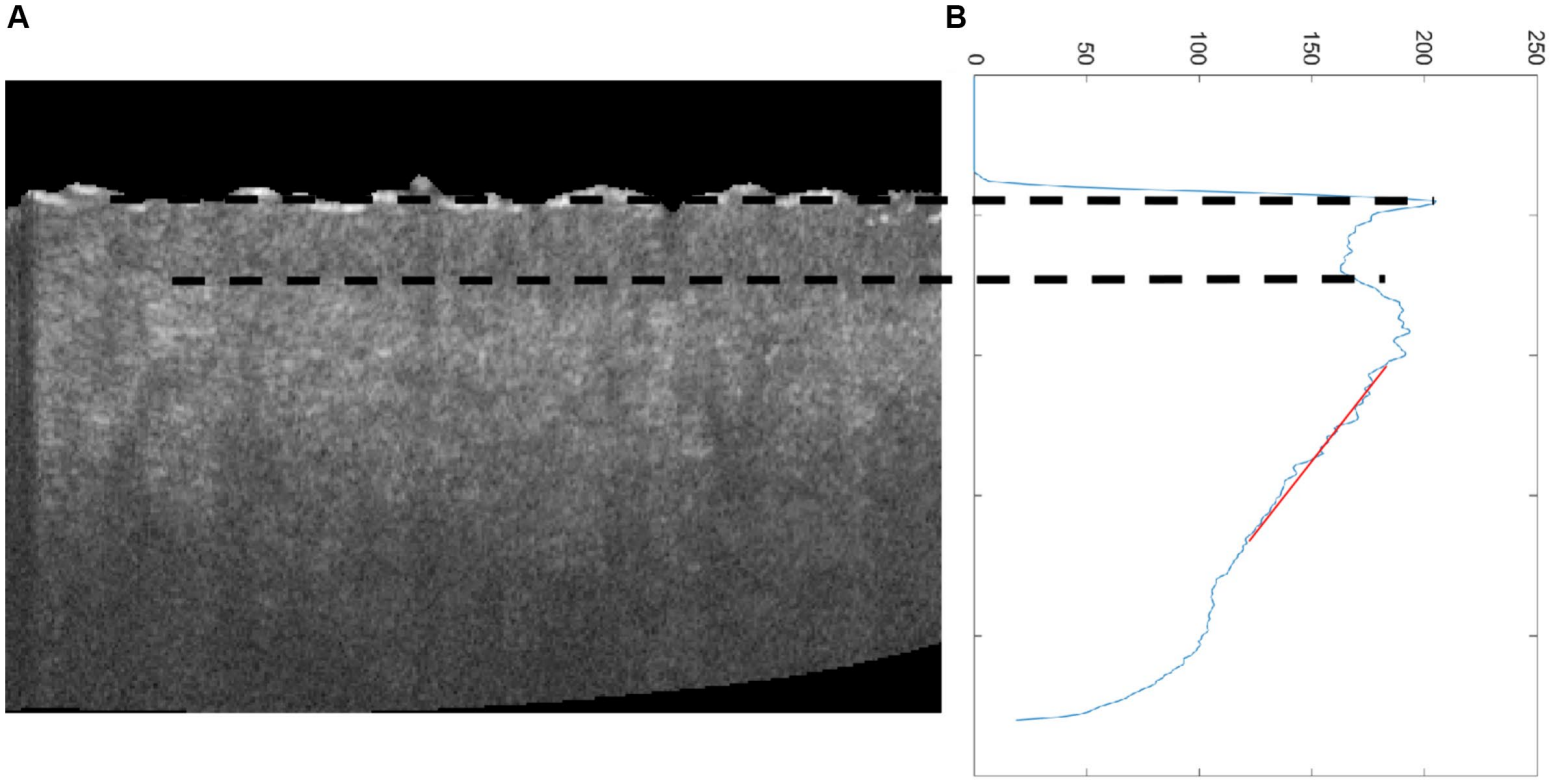
**(A)** Cross-sectional OCT image of skin and **(B)** average OCT signal dependent on depth; the two dot lines are the first peak and valley of average OCT signal, which denotes epidermal thickness, and the red line is the fitting line for estimating attenuation coefficient.

**Figure 10:**
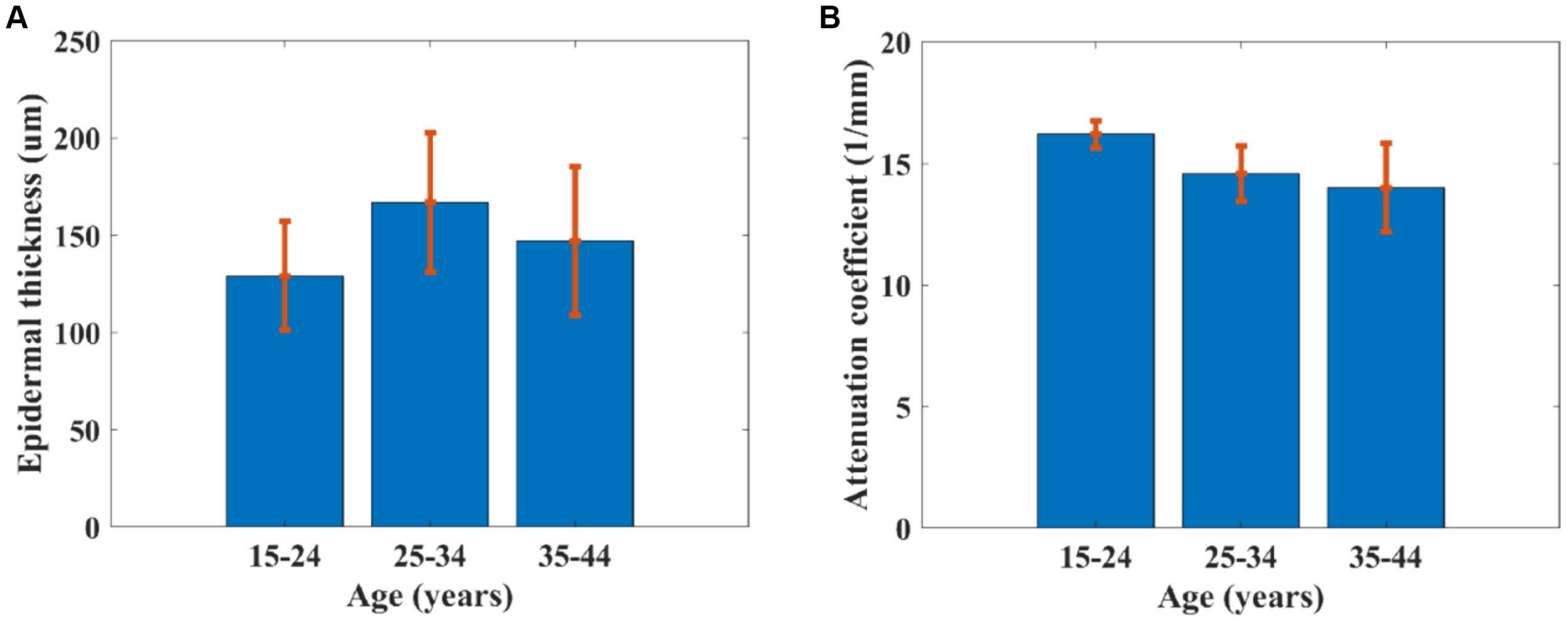
**(A)** Epidermal thickness and **(B)** attenuation coefficient of skin dependent on age.

The advantage of the proposed method in this study for estimating the roughness of skin surface is combined with other parameters such as epidermal thickness (32, 33) and dermal attenuation coefficient (17) based on OCT. Epidermal thickness was estimated based on the interval between the first peak and valley of the average OCT signal in terms of depth, and the attenuation coefficient was calculated based on the fitting line of the OC signal (Figure 9). Figure 10A reveals that the epidermal thickness is not correlated with age, which is consistent with the results found in the previous study (34).

In addition, as shown in Figure 10B, the attenuation coefficient of skin was found to be significantly decreased with increased age, which is consistent with a previous study (17). This is because of a gradual loss of collagen in the skin, resulting in an increase in roughness (2). The phenomenon was also observed in PS-SD-OCT, revealing depth-dependent correlations between the averaged dermal birefringence induced by collagen and the skin roughness parameters of the photoaged skin (35). The skin collagen would be determined using a two-photon confocal imaging for the skin surface (36). However, the image depth of a two-photon confocal image is lower than that of OCT.

Some studies employed traditional image processing techniques, including Gaussian filter, median filter, and differential filter, to emphasize the ideal surface boundary (18). However, these algorithms rely heavily on empirical values for different images. The proposed method in this study is accurate in extracting the surface boundary of skin to overcome the above problem since the CNN can effectively segment the skin surface (16, 22) through large-scale datasets and diverse data augmentation techniques for enhancing the generalization ability of models.

OCT directly measured the height fluctuation of the skin boundary for skin surface roughness, which was quantified by the arithmetic mean roughness and the depth of roughness. Thus, the development of OCT technology can improve the resolution of OCT, which, in turn, improves the accuracy of OCT image segmentation. In addition, the continuous progress in CNN algorithms further enhances the efficiency of the segmentation of skin boundaries.

## 5 Conclusion

In summary, the skin surface roughness is estimated using optical coherence tomography combined with CNN. The experimental results first demonstrated the effectiveness of the proposed algorithm by showing that the calculated value of the arithmetic mean roughness is consistent with the standard value for a roughness standard plate. In addition, the experimental results revealed that the skin surface roughness including the arithmetic mean roughness and depth of roughness depends on age and gender.

The advantage of the proposed method based on OCT is that it can reduce the effect of the skin surface’s natural curvature on roughness and is combined with the epidermal thickness and dermal attenuation coefficient for multi-parameter characterization of skin features. Quantitative assessment of skin features including roughness, epidermal thickness, and attenuation coefficient enables researchers, clinicians, and cosmetic companies to monitor changes in skin condition over time, evaluate the effectiveness of interventions or treatments, and develop targeted products for anti-aging prevention. It serves as a valuable tool in understanding the aging process and developing strategies to maintain and enhance skin health and appearance.

## Data Availability

The original contributions presented in the study are included in the article/supplementary material, further inquiries can be directed to the corresponding authors.
